# Improving the Thermomechanical Properties of Poly(lactic acid) via Reduced Graphene Oxide and Bioderived Poly(decamethylene 2,5-furandicarboxylate)

**DOI:** 10.3390/ma15041316

**Published:** 2022-02-10

**Authors:** Giulia Fredi, Mahdi Karimi Jafari, Andrea Dorigato, Dimitrios N. Bikiaris, Alessandro Pegoretti

**Affiliations:** 1 Department of Industrial Engineering and INSTM Research Unit, University of Trento, Via Sommarive 9, 38123 Trento, Italy; mahdi.karimijafari@studenti.unitn.it (M.K.J.); andrea.dorigato@unitn.it (A.D.); alessandro.pegoretti@unitn.it (A.P.); 2 Laboratory of Polymer Chemistry and Technology, Chemistry Department, Aristotle University of Thessaloniki, 54124 Thessaloniki, Greece; dbic@chem.auth.gr

**Keywords:** nanocomposites, reduced graphene oxide, poly(decamethylene 2,5-furandicarboxylate), furanoate polyesters, polylactic acid, compatibilization

## Abstract

Polylactide (PLA) is the most widely used biopolymer, but its poor ductility and scarce gas barrier properties limit its applications in the packaging field. In this work, for the first time, the properties of PLA solvent-cast films are improved by the addition of a second biopolymer, i.e., poly(decamethylene 2,5-furandicarboxylate) (PDeF), added in a weight fraction of 10 wt%, and a carbon-based nanofiller, i.e., reduced graphene oxide (rGO), added in concentrations of 0.25–2 phr. PLA and PDeF are immiscible, as evidenced by scanning electron microscopy (SEM) and Fourier-transform infrared (FTIR) spectroscopy, with PDeF spheroidal domains showing poor adhesion to PLA. The addition of 0.25 phr of rGO, which preferentially segregates in the PDeF domains, makes them smaller and considerably rougher and improves the interfacial interaction. Differential scanning calorimetry (DSC) confirms the immiscibility of the two polymer phases and highlights that rGO enhances the crystallinity of both polymer phases (especially of PDeF). Thermogravimetric analysis (TGA) highlights the positive impact of rGO and PDeF on the thermal degradation resistance of PLA. Quasi-static tensile tests evidence that adding 10 wt% of PDeF and a small fraction of rGO (0.25 phr) to PLA considerably enhances the strain at break, which raises from 5.3% of neat PLA to 10.0% by adding 10 wt% of PDeF, up to 75.8% by adding also 0.25 phr of rGO, thereby highlighting the compatibilizing role of rGO on this blend. On the other hand, a further increase in rGO concentration decreases the strain at break due to agglomeration but enhances the mechanical stiffness and strength up to an rGO concentration of 1 phr. Overall, these results highlight the positive and synergistic contribution of PDeF and rGO in enhancing the thermomechanical properties of PLA, and the resulting nanocomposites are promising for packaging applications.

## 1. Introduction

Poly(lactic acid) (PLA) is one of the most interesting and widely used biopolymers. PLA is a biodegradable and bioderived thermoplastic linear aliphatic polyester [1,2] widely applied in the packaging and textile fields due to its high elastic modulus (2–3 GPa), good mechanical strength (40–60 MPa), good processability, and high optical transparency [3,4,5]. However, the application of PLA for packaging items is generally circumscribed to rigid thermoformed products, because its scarce strain at break, toughness, and gas permeation properties and its high moisture sensitivity limit its use as a flexible packaging film [2]. Among the techniques to address these drawbacks, one of the most efficient and low-cost methods is to blend PLA with other polymers [6,7]. As the scientific literature demonstrates, PLA-based blends have been prepared with several traditional polymers and biopolymers [8,9,10,11]. Our group has recently blended PLA with several members of an interesting and novel family of biopolymers, i.e., the poly(alkylene 2,5-furandicarboxylate)s (PAFs).

Synthesized via the polycondensation between 2,5-furandicarboxylic acid (FDCA) and an alkylene glycol, PAFs embody the most credible bioderived alternative to fossil-based poly(alkylene terephthalates) (PATs) [12,13,14]. They show thermo-mechanical, gas barrier, and UV-barrier properties comparable with or even superior to those of the corresponding PATs, which highlights their suitability to be applied in the food packaging field [15,16,17,18,19]. Among all PAFs, the most important is arguably poly(ethylene furanoate) (PEF), representing the most convincing bioderived option to poly(ethylene terephthalate) (PET) [20]. Nevertheless, furan-based polyesters have also been synthesized using higher-molecular-weight alkylene glycols with up to 12 carbon atoms [17]. Such PAFs exhibit higher molecular mobility, and therefore lower glass transition and melting temperatures, higher crystallization kinetics, and improved ductility [17].

The existing literature on long-alkyl-chain PAFs mainly targets their synthesis route and thermal properties [17,21,22,23], whereas a small number of works deal with the investigation and optimization of the mechanical and gas barrier properties, and even fewer papers report on the development of PAF-based blends and nanocomposites. Our group has recently added carbon nanotubes (CNTs) to poly(decamethylene furanoate) (PDeF) and investigated the thermo-mechanical properties of the resulting nanocomposites [24]. Moreover, PLA was blended with PAFs that had a variable alkyl chain length (4, 5, 6, 8, 10, or 12 carbon atoms) [25,26,27]. Those works showed that the addition of a small percentage (5–10 wt%) of PAFs into PLA remarkably improved its ductility. This effect was evident especially with odd- or long-alkyl-chain PAFs, whose blends with PLA yielded films with a very interesting combination of physical–mechanical properties [26].

Since all the prepared blends were immiscible and with a relatively poor interfacial interaction, the subsequent step has been the quest for a compatibilizer. Among all the compatibilization techniques, particularly interesting is the nanofiller-induced compatibilization [28,29], where the addition of solid nanoparticles slows down the phase separation, thereby decreasing the domain size and sometimes also improving the interfacial adhesion. Moreover, a suitable selection of the nanofiller can also improve other physical and mechanical properties [7,30,31,32,33]. For example, our group has recently added reduced graphene oxide (rGO) to a blend of PLA and poly(dodecamethylene 2,5-furandicarboxylate) (PDoF). rGO has very interesting physical–mechanical and catalytic properties and is relatively inexpensive, easy to fabricate, and biocompatible [34,35]. In that case, rGO acted as a multifunctional filler, enhancing not only the interfacial adhesion, but also the elastic modulus, the gas barrier properties, and the electrical conductivity [27].

This work aims to exploit the multifunctional role of rGO in a novel bioderived polymer blend, i.e., a PLA/PDeF blend with a PDeF content of 10 wt%. In fact, although the open scientific literature abounds with works on PLA-based blends or nanocomposites, and although PDeF and rGO show great synergic potential in improving the physical–mechanical properties of PLA, no works on such PLA/PDeF/rGO systems can be found in the open scientific literature, to the best of the authors’ knowledge. The PLA/PDeF/rGO nanocomposites have been obtained by solvent casting in the form of thin (50 µm) films, and they have been subsequently characterized microstructurally, thermally, mechanically, and electrically.

## 2. Materials and Methods

### 2.1. Materials

Poly(lactic acid) (PLA)™ Biopolymer 4032D (D-lactic acid content 2%, density 1.24 g/cm^3^, melt flow index (MFI) 7 g/10 min (210 °C, 2.16 kg), melting temperature 155–170 °C) was purchased from NatureWorks LLC (Minnetonka, MN, USA). Poly(1,10-decamethylene 2,5-furandicarboxylate) (PDeF) has been synthesized via a variation of the two-step polycondensation technique from 2,5-dimethylfuran-dicarboxylate and 1,10-decamethylene glycol, as reported in our previous work [36]. rGO was synthesized starting from a concentrated (mg/mL) graphene oxide (GO) water suspension purchased by Graphenea (San Sebastián, Spain), having a declared monolayer content of at least 95% and platelet lateral size of up to 10 µm. Chloroform (HPLC grade, CAS 67-66-3) and hexafluoroisopropanol (HFIP) (RPE grade, CAS 920-66-1) were purchased from Carlo Erba Reagents S.r.l. (Milano, Italy). Hydrazine hydrate (HH) (CAS 10217-52-4) was purchased from Sigma Aldrich (St. Louis, MO, USA).

### 2.2. Sample Preparation

GO was chemically reduced via a reaction in presence of hydrazine hydrate, with a protocol similar to that described in our previous work [27]. Briefly, 20 mL GO solution was added to 180 mL deionized water (DI) in a round-bottom flask. Then, HH was added (HH:GO = 1:1 wt:wt). The suspension was stirred under reflux conditions at 100 °C for 24 h, then left cooling to room temperature, filtered, washed several times with DI, and dried at 50 °C overnight.

**Figure 1:**
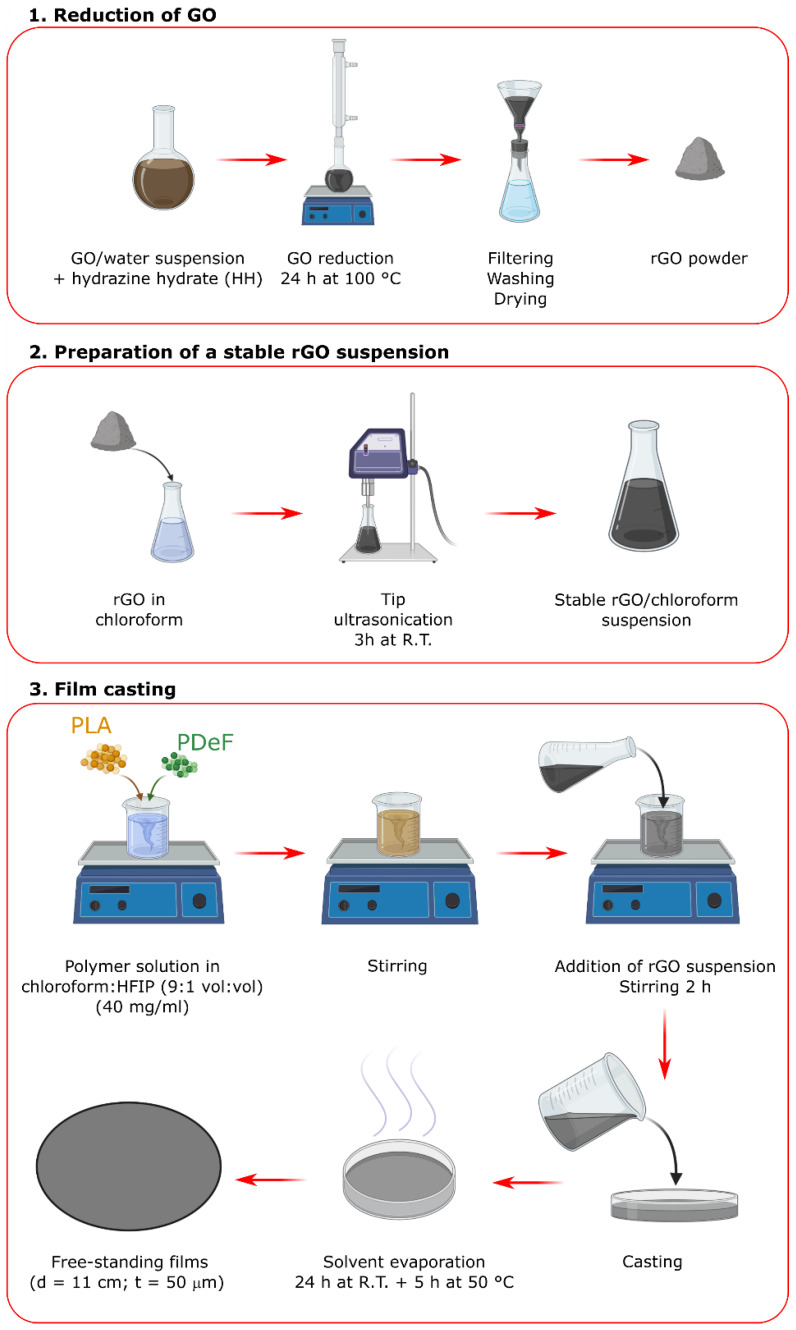
Scheme of the sample preparation route.

The obtained rGO was used as a nanofiller to prepare PLA/PDeF/rGO nanocomposite films via a solvent casting procedure, similarly to what was reported in [27]. The two polymer phases were dried at 50 °C overnight and dissolved in a mixture of chloroform and HFIP (9:1 vol:vol). The concentration of the polymer solution was 4 g in 100 mL solvent. The obtained solutions were stirred at 300 rpm at 50 °C for 2 h, and then a certain amount of rGO suspension was added to the polymer solution, according to the desired rGO concentration in the final nanocomposite film. The rGO suspension was prepared by dispersing a proper amount of rGO in chloroform (1 mg/mL) by tip-ultrasonication (UP-400S, Hielscher Ultrasonics GmbH, Teltow, Germany) for three hours. The volume of rGO suspension added to the polymer solution was varied to reach the desired rGO concentrations in the final nanocomposites. The PLA/PDoF/rGO suspensions were magnetically stirred at 300 rpm at 50 °C for 3 additional hours, then mildly ultrasonicated in a Labsonic LBS1 bath (Falc Instruments Srl, Bergamo, Italy) to remove air bubbles, cast in glass Petri dishes, and dried at R.T. for 24 h and at 50 °C for 5 h. In this way, 50 µm-thick nanocomposite films were prepared with a concentration of rGO varying between 0 and 2 phr. The production process is schematized in Figure 1, while Table 1. lists the prepared films with their nominal weight composition.

### 2.3. Characterization

The microstructure of the prepared films was investigated via the observation of the cryofracture surfaces with a field-emission scanning electron microscope (FE-SEM) Zeiss Supra 60 (Carl Zeiss AG, Oberkochen, Germany), after sputtering with Pt-Pd.

Attenuated total reflectance Fourier-transformed infra-red (ATR-FTIR) spectroscopy was performed on the prepared films with a Perkin-Elmer Spectrum One instrument (Perkin Elmer GmbH, Waltham, MA, USA), in the range 650–4000 cm^−1^, with 100 scans per spectrum (d = 4 cm^−1^).

Differential scanning calorimetry (DSC) was carried out via a Mettler DSC 30 (Mettler Toledo, Inc., Columbus, OH, USA). Specimens (one per composition) of approx. 5 mg were subjected to a first heating scan, a cooling scan, and a second heating scan between −50 and 200 °C at ±10 °C/min, with an N_2_ flow of 100 mL/min. This test led to the measurement of the glass transition, melting, cold crystallization, and crystallization temperatures ($T_{g},\;T_{m},$ $T_{cc},$ $T_{c}$) and enthalpy values ($\Delta H_{m}$, $\Delta H_{cc}$, $\Delta H_{c}$) of both polymer phases. The crystallinity of PLA (${Χ}_{c}^{PLA}$) and PDeF (${Χ}_{c}^{PDeF}$) in the samples were calculated via Equations (1) and (2):

(1) $${Χ}_{c}^{PLA}=\frac{\Delta H_{m}^{PLA}-\Delta H_{cc}^{PLA}}{w^{PLA}\cdot \Delta H_{0}^{PLA}}\cdot 100$$

(2) $${Χ}_{c}^{PDeF}=\frac{\Delta H_{m}^{PDeF}-\Delta H_{cc}^{PDeF}}{w^{PDeF}\cdot \Delta H_{0}^{PDeF}}\cdot 100$$

where $w^{PLA}$ and $w^{PDeF}$ are the PLA and PDeF mass fractions, respectively, and $\Delta H_{0}^{PLA}$ and $\Delta H_{0}^{PDeF}$ are the theoretical melting enthalpy of fully crystalline PLA and PDeF, i.e., 93.7 J/g [37] and 153 J/g [36], respectively.

Thermogravimetric analysis (TGA) was performed with a Q5000IR thermobalance (TA Instruments, Inc., New Castle, DE, USA). Specimens (one per composition) of approx. 4 mg were tested up to 700 °C at 10 °C/min, under a nitrogen flow of 10 mL/min. These tests led to the measurement of the residual mass at 150 °C after the complete removal of the residual solvent ($m_{150^{\circ }C}$), the onset temperature of degradation, determined with the tangent method ($T_{onset}$), and the peak degradation temperature, corresponding to the peak of the mass loss derivative (DTG) curve ($T_{d}$).

Quasi-static tensile tests were carried out with a universal testing machine Instron 5969 (Instron, Norwood, MA, USA), equipped with a 100 N load cell. Rectangular specimens (at least five per composition) with nominal in-plane dimensions of 80 × 5 mm^2^ were glued onto paper frames to ease their handling, mounted on the testing machine with a gauge length of 50 mm, and tested at 10 mm/min. In this way, the most important mechanical parameters were determined, i.e., the elastic modulus (E), calculated as the slope of the stress–strain curve in the initial linear region, the stress and the strain at yield ($\sigma_{y}$, $\varepsilon_{y}$), and the stress and strain at break $(\sigma_{b}$, $\varepsilon_{b}$).

Finally, electrical resistivity was measured with a four-point test on rectangular specimens with in-plane dimensions of 10 × 50 mm^2^, following the ASTMD4496-04 standard. A DC voltage generator ISO-Tech IPS 303DD (Milano, Italy) was connected to the specimens, an ammeter was connected in series, and a voltmeter was connected in parallel to the two inner electrodes, placed at a distance of 3.69 mm. The volume resistivity ρ was calculated via Equation (3):

(3) $$\rho =R\frac{w\cdot t}{l}$$

where R is the resistance calculated as the slope of the voltage–current plot, linear in the measurement range, w and t are the specimen width and the thickness, respectively, and l is the distance between the inner electrodes. This configuration allows the measurement of resistivity values up to 10^7^ Ω·cm, while the resistivity of more insulating films was determined with a Keithley 6517A electrometer/high-resistance meter (Cleveland, OH, USA) and an 8009 resistivity test fixture, following the ASTM D257 standard. A constant voltage of 50 V was applied to circular samples with a nominal diameter of 70 mm.

## 3. Results and Discussion

**Figure 2:**
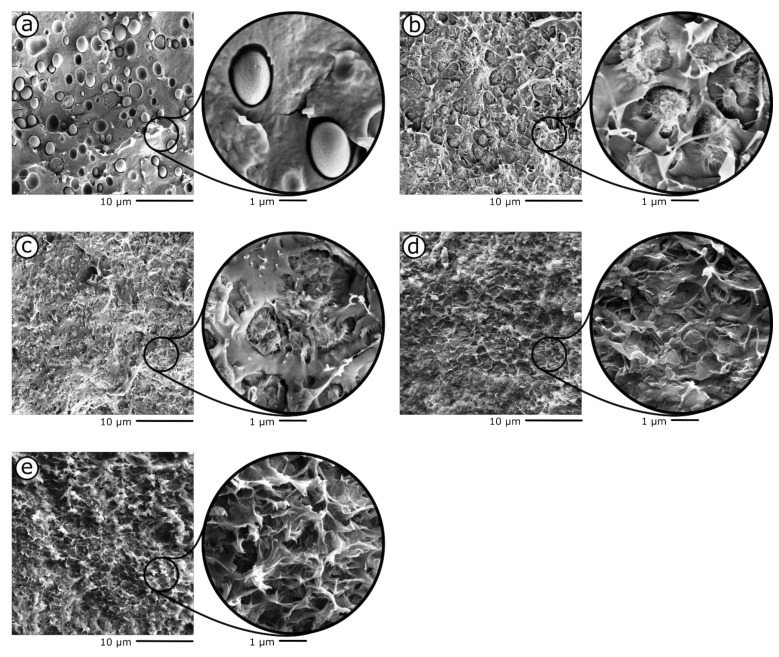
SEM micrographs of the cryofracture surface of the prepared films. (**a**) PLA-PDeF10; (**b**) PLA-PDeF10-rGO0.25; (**c**) PLA-PDeF10-rGO0.5; (**d**) PLA-PDeF10-rGO1; (**e**) PLA-PDeF10-rGO2.

Figure 2a–e shows the SEM micrographs of the cryofracture surface of the samples PLA-rGO10 and PLA-PDeF10-rGOx (x = 0.25–2 phr). As already observed for other PLA/PAF blends [26,27,38,39,40], in this case the blend is also immiscible (Figure 2a), and PDeF forms smooth spheroidal domains with a poor interfacial adhesion with PLA and an average size of 1.9 ± 0.3 µm. The addition of rGO considerably modifies the appearance of the fracture surface. As observable from Figure 2b, adding 0.25 phr of rGO reduces the PDeF domain size (1.4 ± 0.3 µm) while increasing their roughness and their interfacial interaction with the surrounding PLA matrix, which suggests improved compatibility. An increase in the rGO concentration (Figure 2c) further modifies the morphology of the fracture surface and, at the highest rGO concentrations (Figure 2d,e), the PDeF domains are no longer visible. In fact, similarly to what was observed in our previous work on PLA/PDoF/rGO nanocomposites, the rGO is preferentially distributed in the PDeF domains, which can accommodate most of the rGO until a certain nanofiller content (0.5 phr). Above this value, the rGO is partially accommodated in the PLA matrix.

**Figure 3:**
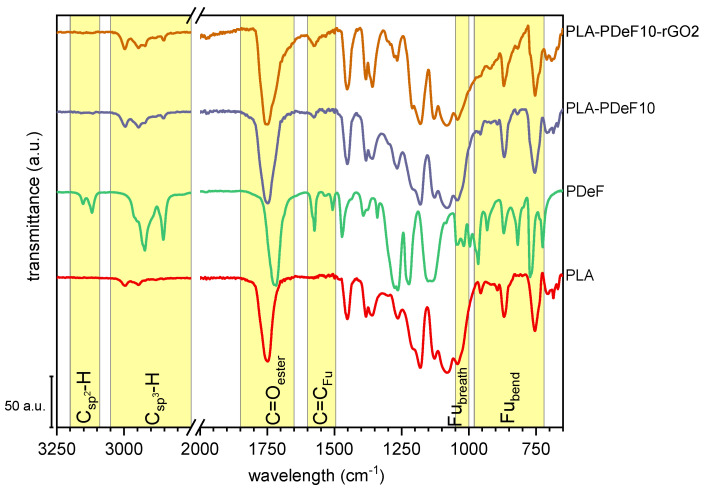
Representative ATR-FTIR spectra (baseline corrected and vertically translated) of the prepared samples.

The FTIR spectra of the prepared films are presented in Figure 3, which includes the spectra of some selected compositions after baseline correction, normalization to the most intense band, and vertical shifting. As already observed in our previous works [26], neat PLA shows the stretching of C–H at 2950–3000 cm^−1^, C=O (1751 cm^−1^), and C–O–C (1180 cm^−1^) [41], as well as the contributions of amorphous and crystalline regions, found at 869 and 755 cm^−1^, respectively [42,43,44]. PDeF shows all bands of PAFs, i.e., the symmetrical and asymmetrical stretching of the furan ring (3119 and 3152 cm^−1^), the symmetrical and asymmetrical C–H stretching of the methylene groups of the alkyl chain (2920 and 2850 cm^−1^), the signals of the furan C=C (1574 and 1530 cm^−1^) [45,46], the ester C=O stretching (1717 cm^−1^) [47,48], the furan ring breathing (1018 cm^−1^) and bending (966, 820, and 772 cm^−1^).

The spectra of PLA-PDeF10 and PLA-PDeF10-rGO2, also reported in Figure 3, are very similar to that of PLA, which could be expected given the reduced weight fraction of PDeF and rGO. The occurrence of PDoF can be inferred by an increase in the intensity of the bands at 2920 and 2850 cm^−1^, which is related to the C–H stretching of the alkyl methylene groups and the asymmetry of the C=O band (1751 cm^−1^). Conversely, the rGO does not contribute to the FTIR spectra with any additional vibrations, which could be expected, given the low rGO concentration and the featurelessness of its ATR-FTIR spectrum [7]. Neither PDeF nor rGO significantly affects the bands at 869 and 755 cm^−1^, relative to PLA’s amorphous and crystalline regions, respectively, as neither the position nor the intensity of these bands shows a clear trend with the sample composition.

**Figure 4:**
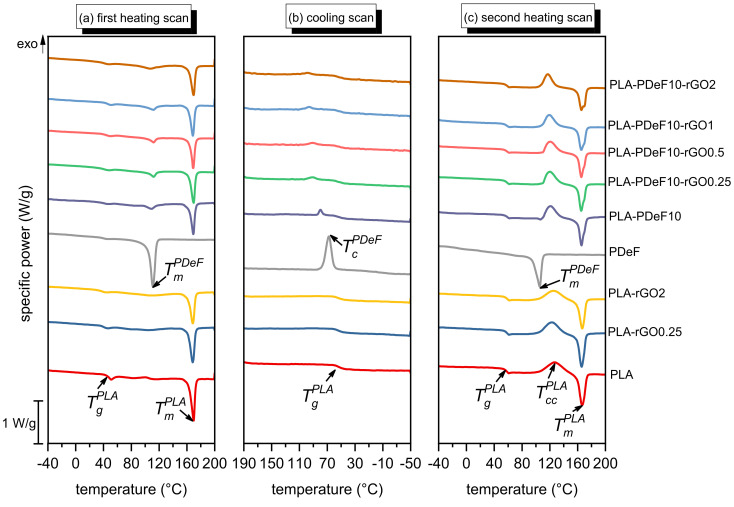
DSC thermograms of the prepared samples (**a**) first heating scan; (**b**) cooling scan; (**c**) second heating scan.

Figure 4a–c shows the DSC thermograms of all the prepared samples, and Table 2 collects the most important DSC results. Neat PLA undergoes glass transition at 40.9 °C in the first heating scan (Figure 4a), while the $T_{g}$ increases in the second heating scan (57.4 °C) due to the removal of solvent (chloroform and/or HFIP) that acts as a plasticizer, as observed in previous works about PLA-based solvent cast films [27]. Neat PLA also shows a melting peak at 169.4 °C and, in the second heating scan, also a cold-crystallization peak at 126.1 °C, not visible in the first heating scan. In fact, the crystallinity of neat PLA is 41.3% in the first heating scan and only 2.3% in the second heating scan, since the processing conditions, i.e., slow solvent evaporation and subsequent thermal treatment 5 h at 50 °C, favor high-crystallinity degrees.

The nanocomposites PLA-rGOx present very similar thermograms as that of neat PLA, as the effect on the $T_{g}$, $T_{m}$, and $T_{cc}$ is almost negligible. Conversely, a small amount of rGO strongly enhances the crystallinity of PLA in the second heating scan, which increases from 2.3% of neat PLA up to 5.2% of PLA-rGO0.25. Instead, no further substantial increase is observable for PLA-rGO2 (${Χ}_{c}^{PLA}$ = 5.4%) since, above a certain threshold, nucleation competes with the chain mobility restriction [49].

Neat PDeF shows a melting peak at approx. 110 °C in both heating scans and a crystallization peak at 68.7 °C in the cooling scan, which evidences the faster crystallization kinetics of PDeF compared to PLA. The measured phase change temperatures are in good agreement with those found by Tsanaktsis et al. [36] on neat PDeF. The crystallinity of PDeF is 51.4% in the first heating scan and 32.1% in the second heating scan. Instead, the $T_{g}$, which should be located at approx. 1 °C [36], is not detectable, probably due to the sensitivity limits of the instrument.

The blend PLA-PDeF10 shows the thermal transitions of both polymer phases. The $T_{g}$ of PLA is not remarkably different from that measured on neat PLA, which accounts for the immiscibility of the prepared blend, in good agreement with FTIR and SEM analyses. The addition of rGO into PLA/PDeF blends increases the $T_{c}^{PDeF}$, from 68.4 °C of neat PDeF, to 80.1 °C of PLA-PDeF10, up to 98.6 °C of PLA-PDeF10-rGO2, thereby anticipating the crystallization event. This supports the hypothesis that rGO is preferentially distributed in PDeF domains, as observable in the SEM micrographs (Figure 2).

The degree of crystallinity of PLA measured in the first heating scan is quite high and comparable among the prepared samples (35–40%), in good agreement with the results of FTIR. On the other hand, in the second heating scan, the crystallinity degree of PLA is considerably lower (2–5%), without any specific trends with the amount of rGO for the samples PLA-PDeF10-rGOx. The addition of rGO promotes the crystallization of PLA, as discussed before, while the addition of PDeF does not have any remarkable effect. The crystallinity degree of the PDeF phase in the first heating scan, already quite high for neat PDeF (51.4%) and for the sample PLA-PDeF10 (45.8%), further increases with rGO addition. The maximum value is 72.0%, found for the sample PLA-PDeF10-rGO1. Unfortunately, the value of $X_{C}^{PDeF}$ in the second heating scan cannot be calculated because of the superposition of the PDeF melting signal with the PLA cold crystallization peak. Therefore, it is not possible to draw information about the effect of PLA and rGO on the crystallinity of PDeF from the melt.

The simultaneous addition of PDoF and rGO shifts the cold crystallization peak to lower temperatures and slightly increases $X_{C}^{PLA}$, especially at higher rGO concentrations, which is also a sign of the increased crystallization kinetics in the solid state. Moreover, in the samples PLA-PDeF10-rGOx, PLA manifests a double melting behavior, observed previously in the literature [27,50] and attributed to the melting of two types of crystallites: the bigger ones, formed during melt crystallization, and the smaller, originated during cold crystallization.

**Figure 5:**
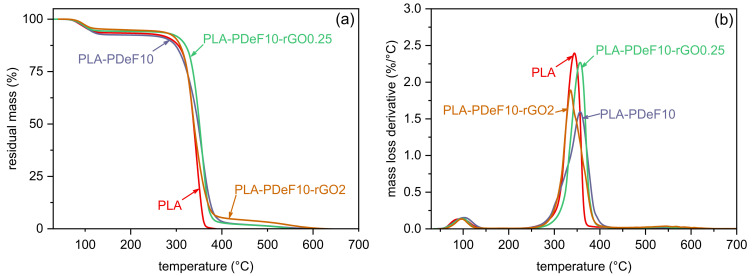
TGA thermograms of the prepared samples. Residual mass (**a**) and mass loss derivative (**b**) as a function of temperature.

TGA tests were performed to investigate the effect of PDeF and rGO on the thermal degradation resistance of PLA. Figure 5a,b shows representative TGA thermograms of some selected compositions, while the most important TGA results are collected in Table 3. All samples exhibit a first mass loss of 5–7 wt% at approx. 100 °C, related to the evaporation of the residual solvent, in good agreement with DSC results. The presence of residual solvent in PLA-based films prepared via solvent casting from chloroform-based solutions is a well-known problem and it was also found in previous works of our group [26,27]. Nevertheless, since the amount of residual solvent is similar across all compositions, it is reasonable to suppose that the differences in thermomechanical properties found with the present characterization truly reflect the different material properties. After the solvent evaporation, the degradation of neat PLA begins at approx. 320 °C ($T_{onset}$, Table 3) and the maximum degradation rate is found at 344 °C. The addition of rGO improves the thermal degradation resistance of PLA, by shifting both $T_{onset}$ and $T_{d}$ to higher temperatures. On the other hand, the addition of PDeF does not remarkably modify the degradation behavior of PLA.

**Figure 6:**
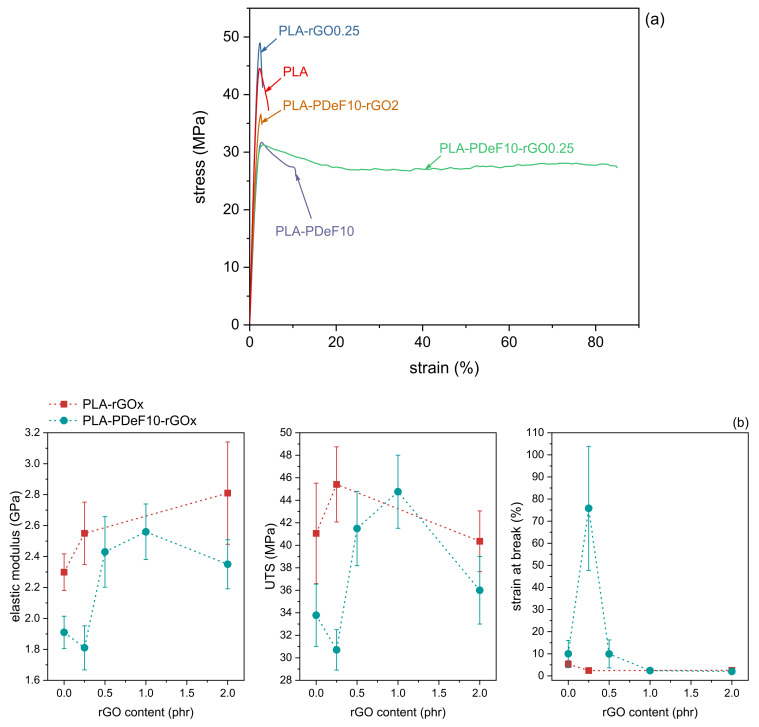
Results of the tensile tests on the prepared samples. (**a**) Representative stress–strain curves; (**b**) elastic modulus, maximum stress, and strain at break as a function of the rGO content.

Figure 6a,b illustrates the results of the tensile tests on the prepared films. Figure 6a shows representative stress–strain curves of some selected samples, i.e., PLA, PLA-rGO0.25, PLA-PDeF10, PLA-PDeF10-rGO0.25, and PLA-PDeF10-rGO2. Figure 6b reports the main results of the tensile tests, i.e., elastic modulus (E), ultimate tensile stress (UTS), and strain at break ($\varepsilon_{b}$) as a function of the rGO concentration The UTS, determined in correspondence of the maximum stress, has been selected as an indication of the tensile strength because the various compositions do not show a uniform mechanical behavior. In fact, some compositions show an evident yield point, while others break before yielding, and therefore it is not meaningful nor possible to draw conclusions about the mechanical behavior by comparing the yield or the break strength.

The results of PLA and PLA-rGOx samples evidence the positive contribution of a little amount of rGO on the mechanical properties of PLA. The sample PLA-rGO0.25 shows improved elastic modulus (+11%) and UTS (+11%) compared to neat PLA, but lower strain at break (−54%), similarly to that which can be found in the literature for similar graphene-based nanocomposites [51]. Increasing the rGO concentration leads to further embrittlement: the sample PLA-rGO2 fails before yielding, probably due to excessive nanofiller agglomeration, which also leads to a decrease in the UTS compared to the sample PLA-rGO0.25, comparable to that which is found in the literature for similar systems [49].

The addition of PDeF to PLA decreases its elastic modulus (−16%) and UTS (−17%) and increases the strain at break (+92%). Even though the data dispersion of the strain at break results is not negligible, these findings are in good agreement with previous results on PLA/PAF blends [26,27], which highlighted the positive contribution of a small amount of PAFs with a long aliphatic chain on the ductility of PLA. What is even more remarkable is the impact of the addition of 0.25 phr of rGO to this blend. The sample PLA-PDeF10-rGO0.25 shows similar values of elastic modulus and UTS as the sample PLA-PDeF10, but a significantly higher $\varepsilon_{b}$, which rises to 76 ± 28%, a clear indication that rGO is effective in providing blend compatibilization [7]. Further rGO addition strongly impairs the ductility but promotes an increase in elastic modulus and UTS, which are both maximized for PLA-PDeF10-rGO1.

In summary, these results indicate a positive synergistic effect of PDeF and rGO in improving the ductility of PLA. According to the requirements of the specific applications, the compositions with the best combination of properties are PLA-PDeF10-rGO0.25, to maximize the ductility, and PLA-PDeF10-rGO0.5, to have a balanced property set, with elastic modulus and UTS comparable with those of PLA and higher strain at break.

**Figure 7:**
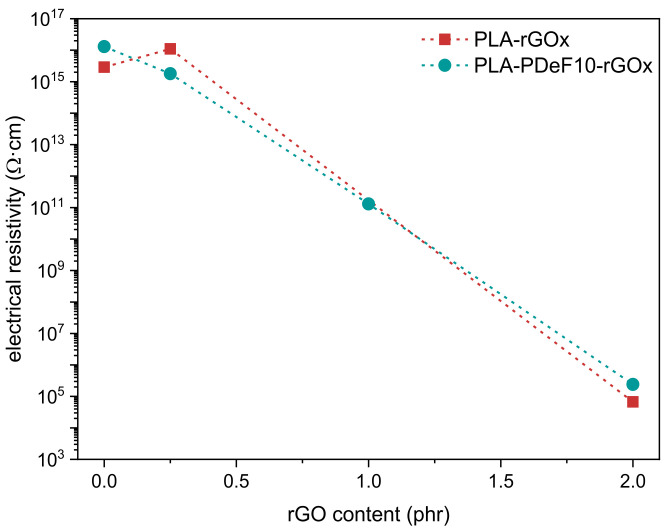
Electrical resistivity of the prepared samples (Log scale) as a function of the rGO content.

Figure 7 reports the electrical resistivity (ρ) of the prepared samples as a function of the rGO content. Adding 0.25 phr of rGO does not significantly decrease the resistivity neither of PLA nor of PLA-PDeF10. To see an appreciable increase in conductivity and move from insulative (ρ higher than 10^11^ Ω·cm, following the ANSI/EIA-541 standard “Packaging Materials Standards for electrostatic discharge (ESD) sensitive Items”) to dissipative samples (ρ between 10^4^ and 10^11^ Ω·cm), the rGO concentration must increase up to 2 phr. This percolation threshold is considerably higher than that identified in other literature works on carbon- and graphene-based nanocomposites, generally between 0.1 and 0.5 wt% [30,51,52], but it is in line with what was found for PLA-PDoF10-rGOx samples [27]. Similar to that which was reported in that work, the reason behind such a high percolation threshold could stem from the small size, wrinkled morphology, and poor dispersion of these rGO sheets.

## 4. Conclusions

This work explored the role of PDeF (10 wt%) and rGO (0.25–2 phr), alone and combined, on improving the thermomechanical properties of PLA films prepared by solvent casting. SEM micrographs evidenced that PDeF and PLA were immiscible, as also suggested by FTIR, and PDeF was present as spheroidal domains with a smooth appearance and a poor interfacial interaction with PLA. The addition of a small amount (0.25 phr) of rGO, which segregated in the PDeF domains, remarkably changed their appearance, as they became rougher and smaller, and the interfacial interaction considerably increased.

This effect was likely at the basis of the considerable increase in strain at break of the sample PLA-PDeF10-rGO0.25 compared to neat PLA and PLA-PDeF10. In fact, the addition of 10 wt% of PDeF already improved PLA’s ductility and increased its strain at break from 5.4 to 10.0%. However, the compatibilizing effect played by rGO further enhanced the ductility of the resulting films, which reached an average value of 76%. Further additions of rGO brought the $\varepsilon_{b}$ back to values lower than 10%, due to excessive agglomeration and embrittlement, but increased the elastic modulus and mechanical strength. For instance, the sample PLA-PDeF10-rGO0.5 exhibited an average elastic modulus of 2.30 GPa, an average tensile strength of 41 MPa, and an average strain at break of 9.9%, thereby retaining the mechanical stiffness and strength of PLA and improving the ductility.

PDeF and rGO also improved the crystallization kinetics and especially the thermal degradation resistance of PLA, by improving both $T_{onset}$ and $T_{d}$, due to the higher thermal degradation resistance of PDeF compared to PLA and the role played by the rGO nanofiller network. Additionally, the prepared films resulted to be electrically dissipative (electrical resistivity of approx. 10^5^ Ω·cm) with an rGO concentration of 2 phr, regardless of the presence of PDeF.

Overall, this work showed the positive impact of PDeF and rGO on the properties of PLA and produced nanocomposite films with a property set that is very promising for packaging applications.

## Figures and Tables

**Table 1:** List of prepared samples with nominal weight composition.

Sample	PLA (wt%) *	PDeF (wt%) *	rGO (phr) **
PLA	100	0	0
PLA-rGO0.25	100	0	0.25
PLA-rGO2	100	0	2
PLA-PDeF10	90	10	0
PLA-PDeF10-rGO0.25	90	10	0.25
PLA-PDeF10-rGO0.5	90	10	0.5
PLA-PDeF10-rGO1	90	10	1
PLA-PDeF10-rGO2	90	10	2

* Weight fractions of PLA and PDeF sum up to 100%; ** phr = parts per hundred resin (grams every 100 g of total polymer mass (PLA + PDeF)).

**Table 2:** Main results of the DSC tests on the prepared samples.

		PLA	PLA-rGO0.25	PLA-rGO2	PDeF	PLA-PDeF10	PLA-PDeF10-rGO0.25	PLA-PDeF10-rGO0.5	PLA-PDeF10-rGO1	PLA-PDeF10-rGO2
h1	$T_{g}^{PLA}$ (°C)	40.9	40.0	39.2	–	41.5	40.8	43.3	45.8	40.6
	$T_{m}^{PDeF}$ (°C)	–	–	–	110.2	109.1	111.8	111.9	111.9	106.9
	$\Delta H_{m}^{PDeF}$ (J/g)	–	–	–	78.6	7.0	8.8	6.9	10.9	10.0
	$T_{m}^{PLA}$ (°C)	169.4	168.1	168.3	–	168.4	169.2	169.8	169.0	169.0
	$\Delta H_{m}^{PLA}$ (J/g)	38.7	36.1	33.1	–	29.4	30.7	27.8	28.9	30.6
	$X_{C}^{PLA}$ (%)	41.3	38.6	36.0	–	34.9	36.4	33.0	34.3	36.2
	$X_{C}^{PDeF}$ (%)	–	–	–	51.4	45.8	57.7	45.3	72.0	66.7
c	$T_{c}^{PDeF}$ (°C)	–	–	–	68.4	80.1	90.7	91.6	96.7	98.6
	$\Delta H_{c}^{PDeF}$ (J/g)	–	–	–	50.1	4.2	4.0	4.9	5.8	4.7
h2	$T_{g}^{PLA}$ (°C)	57.4	57.9	57.9	–	58.5	58.5	58.1	57.9	58.1
	$T_{m}^{PDeF}$ (°C)	–	–	–	110.4	–	–	–	–	–
	$\Delta H_{m}^{PDeF}$ (J/g)	–	–	–	49.1	–	–	–	–	–
	$T_{cc}^{PLA}$ (°C)	126.1	122.1	125.1	–	120.9	119.9	120.2	119.1	117.1
	$\Delta H_{cc}^{PLA}$ (J/g)	38.0	37.7	32.7	–	32.1	30.4	25.6	29.5	26.7
	$T_{m}^{PLA}$ (°C)	166.1	165.4	166.4	–	165.5	164.5	164.6	164.8	165.1
	$\Delta H_{m}^{PLA}$ (J/g)	40.2	42.6	37.7	–	34.1	32.1	28.9	30.5	31.2
	$X_{C}^{PLA}$ (%)	2.3	5.2	5.4	–	2.4	2.0	3.9	1.2	5.3
	$X_{C}^{PDeF}$ (%)	–	–	–	32.1	–	–	–	–	–

h1 = first heating scan; c = cooling scan; h2 = second heating scan;  ${Χ}_{c}^{PLA}$ = crystallinity of PLA; ${Χ}_{c}^{PDeF}$ = crystallinity of PDeF; $T_{g}^{PDeF}$ = glass transition temperature of PDeF; $T_{g}^{PLA}$ = glass transition temperature of PLA; $T_{m}^{PDeF}$ = melting temperature of PDeF; $\Delta H_{m}^{PDeF}$ = melting enthalpy of PDeF; $T_{m}^{PLA}$ = melting temperature of PLA; $\Delta H_{m}^{PLA}$ = melting enthalpy of PLA; $T_{c}^{PDeF}$ = crystallization temperature of PDeF; $\Delta H_{c}^{PDeF}$ = crystallization enthalpy of PDeF; $T_{cc}^{PLA}$ = cold crystallization temperature of PLA; $\Delta H_{cc}^{PLA}$ = cold crystallization enthalpy of PLA; – = not detectable.

**Table 3:** Main results of the TGA tests on the prepared samples.

Sample	$m_{150^{\circ }C}\;(\%)$	$T_{onset}\;{(}^{\circ }C)$	$T_{d}\;(\%)$
PLA	93.9	320.5	343.9
PLA-rGO0.25	95.6	329.5	362.5
PLA-rGO2	94.5	331.4	360.9
PLA-PDeF10	94.8	324.9	340.8
PLA-PDeF10-rGO0.25	95.1	322.6	346.0
PLA-PDeF10-rGO0.5	95.2	315.3	352.1
PLA-PDeF10-rGO1	93.9	320.5	343.9
PLA-PDeF10-rGO2	92.6	315.4	356.7

$m_{150^{\circ }C}$ = residual mass at 150 °C; $T_{onset}$ = onset degradation temperature; $T_{d}$ = degradation temperature (peak of the mass loss derivative signal).

## Data Availability

The data supporting the findings of this work are available on request from the corresponding author.
